# IL-25/IL-33–responsive T_H_2 cells characterize nasal polyps with a default T_H_17 signature in nasal mucosa

**DOI:** 10.1016/j.jaci.2015.10.019

**Published:** 2016-05

**Authors:** Emily P.S. Lam, Harsha H. Kariyawasam, Batika M.J. Rana, Stephen R. Durham, Andrew N.J. McKenzie, Nicholas Powell, Nara Orban, Melissa Lennartz-Walker, Claire Hopkins, Sun Ying, Joanne Rimmer, Valerie J. Lund, David J. Cousins, Stephen J. Till

**Affiliations:** a Division of Asthma, Allergy and Lung Biology, Guy's Hospital, King's College London, London, United Kingdom; b Allergy and Medical Rhinology Section, Royal National Throat Nose Ear Hospital, University College London, London, United Kingdom; c Medical Research Council Laboratory of Molecular Biology, Cambridge, United Kingdom; d Section of Allergy and Clinical Immunology, National Heart and Lung Institute, Imperial College London, London, United Kingdom; e Division of Transplantation Immunology and Mucosal Biology and Medical Research Council Centre for Transplantation, King's College London, London, United Kingdom; f Department of Infection, Immunity and Inflammation, NIHR Leicester Respiratory Biomedical Research Unit, Leicester Institute for Lung Health, University of Leicester, Leicester, United Kingdom; g Department of ENT, Guy's and St Thomas' Hospital, London, United Kingdom; h Medical Research Council and Asthma UK Centre in Allergic Mechanisms of Asthma, London, United Kingdom

**Keywords:** Chronic rhinosinusitis with nasal polyps, nasal mucosa, IL-25, IL-33, IL-17RB, ST2, T-cell phenotype, T_H_2 cells, T_H_17 cells, T-cell receptor Vβ repertoire, microarray, AIM2, Absent in melanoma 2, CDR3, Complementarity-determining region 3, CRSwNP, Chronic rhinosinusitis with nasal polyposis, CRTH2, Chemoattractant receptor-homologous molecule expressed on T_H_2 cells, ILC2, Type 2 innate lymphoid cell, TCR Vβ, T-cell receptor variable β-chain

## Abstract

**Background:**

Chronic rhinosinusitis with nasal polyposis (CRSwNP) in Western countries is characterized by eosinophilia, IgE production, and T_H_2 cytokine expression. Type 2 innate lymphoid cells from polyps produce IL-5 and IL-13 in response to IL-25 and IL-33, although the relevance of this axis to local mucosal T-cell responses is unknown.

**Objective:**

We sought to investigate the role of the IL-25/IL-33 axis in local mucosal T-cell responses in patients with CRSwNP.

**Methods:**

Polyp tissue and blood were obtained from patients undergoing nasal polypectomy. Control nasal biopsy specimens and blood were obtained from healthy volunteers. Tissue was cultured in a short-term explant model. T-cell surface phenotype/intracellular cytokines were assessed by means of flow cytometry. T-cell receptor variable β-chain analysis was performed with the immunoSEQ assay. Microarrays were performed for gene expression analysis.

**Results:**

IL-25 receptor (IL-17RB)–expressing T_H_2 effector cells were identified in nasal polyp tissue but not the healthy nasal mucosa or periphery. IL-17RB^+^CD4^+^ polyp–derived T_H_2 cells coexpressed ST2 (IL-33 receptor) and responded to IL-25 and IL-33 with enhanced IL-5 and IL-13 production. Within IL-17RB^+^CD4^+^ T cells, several identical T-cell receptor variable β-chain complementarity-determining region 3 sequences were identified in different subjects, suggesting clonal expansion driven by a common antigen. Abundant IL-17–producing T cells were observed in both healthy nasal mucosal and polyp populations, with T_H_17-related genes the most overexpressed compared with peripheral blood T cells.

**Conclusion:**

IL-25 and IL-33 can interact locally with IL-17RB^+^ST2^+^ polyp T cells to augment T_H_2 responses in patients with CRSwNP. A local T_H_17 response might be important in healthy nasal mucosal immune homeostasis.

Chronic rhinosinusitis with nasal polyposis (CRSwNP) is an umbrella term for a heterogeneous group of inflammatory disorders characterized by persistent polypoid inflammation of the sinonasal mucosa (≥12 weeks) and nasal obstruction.^1^ Symptoms are often severe and only partially responsive to treatment, and disease is commonly associated with difficult-to-treat asthma.^1^, ^2^ There is an urgent unmet clinical need to understand the immunopathology of CRSwNP. Several studies have indicated regional variation in CRSwNP endotypes. Western countries show a predominance of eosinophilic T_H_2-associated polyps, and *Staphylococcus aureus* superantigens have been implicated in driving the T_H_2 response.^3^, ^4^, ^5^ Conversely, CRSwNP in patients from southern Asia is associated with neutrophilic infiltration and a local T_H_1/T_H_17 signature.^3^, ^4^, ^6^ Although potential sources of proeosinophilic cytokines in patients with CRSwNP include T cells, type 2 innate lymphoid cells (ILC2s), mast cells, and eosinophils, the local immune mechanisms regulating cytokine production remain poorly understood. Relatively little is also known of T-cell responses in the healthy nasal mucosa, although the local microenvironment appears to suppress T_H_2 responses.^7^

Recently, the epithelial cell–derived cytokines IL-25 and IL-33, acting through their respective receptors IL-17RB and ST2, have been implicated in promoting T_H_2 responses in animal models of allergic inflammation.^8^, ^9^, ^10^ Expression of IL-17RB has been demonstrated on human peripheral blood T_H_2 cells differentiated *in vitro* by thymic stromal lymphopoietin–treated dendritic cells and on freshly isolated CD4^+^ T cells from patients with Churg-Strauss syndrome.^11^, ^12^ IL-25 is also expressed within the bronchial mucosa of asthmatic patients and in the skin during allergen-induced late responses.^11^, ^13^ Furthermore, ILC2s coexpress IL-17RB and ST2 and produce IL-5 and IL-13 in response to IL-25 and IL-33.^14^, ^15^ ST2 is associated with T_H_2 immune responses in mice,^16^, ^17^ and expression is increased in ILC2s and eosinophils from patients with CRSwNP.^18^, ^19^, ^20^ In human subjects baseline levels of IL-33 mRNA in epithelial cells derived from treatment-recalcitrant nasal polyps are increased compared with levels in cells derived from treatment-responsive nasal polyps.^21^ However, the local mucosal T-cell response in patients with CRSwNP and the potential interaction of T cells in the nasal mucosa with IL-25 or IL-33 have not been explored.

Therefore we hypothesized that the IL-25/IL-33 axis is involved in directing local mucosal T_H_2 responses in patients with eosinophilic CRSwNP. To test this hypothesis, we extensively phenotyped nasal T-cell responses from tissue explants of patients with CRSwNP and healthy control subjects.

## Methods

Detailed methods used in this study and reagent sources can be found in the Methods section in this article's Online Repository at www.jacionline.org. Clinical and demographic data for patients with CRSwNP and healthy volunteers are shown in Table E1 in this article's Online Repository at www.jacionline.org.

## Results

### Nasal polyp explant T cells are of an effector memory phenotype

**Fig E1:**
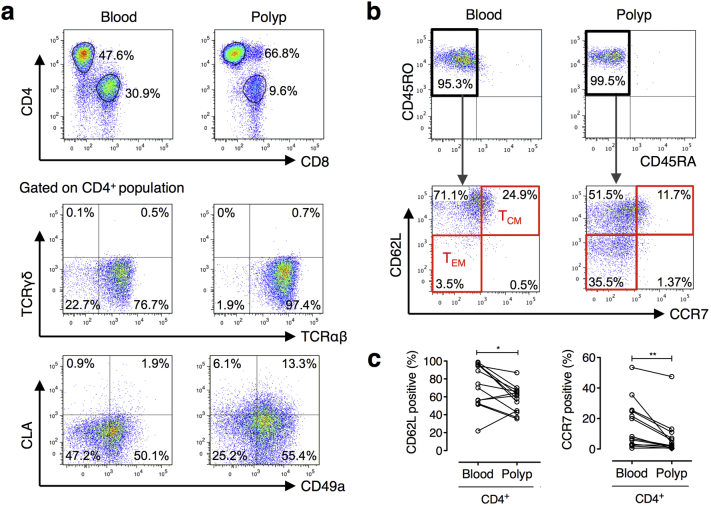


The majority of donor-matched polyp- and peripheral blood–derived CD4^+^ and CD8^+^ T cells were determined to be αβ T cells. γδ T cells formed a minimal proportion of the T-cell population (see Fig E1 and Table E2 in this article's Online Repository at www.jacionline.org). After short-term culture, both polyp and blood populations expressed high levels of CD45RO, which is consistent with a memory phenotype after restimulation. The majority of T cells in polyp cultures expressed significantly less CD62 ligand and CCR7 compared with blood T cells and displayed higher expression of CD49a, an integrin expressed by tissue-resident memory cells,^22^, ^23^ suggesting that nasal polyp–derived T cells were predominately of an effector memory phenotype.^24^

### T_H_17 and T_H_2 cytokine profiles are detected in nasal polyps

**Fig E2:**
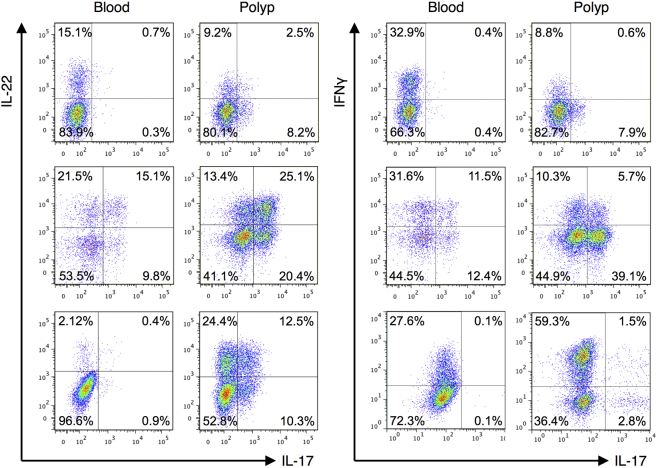


**Fig 1:**
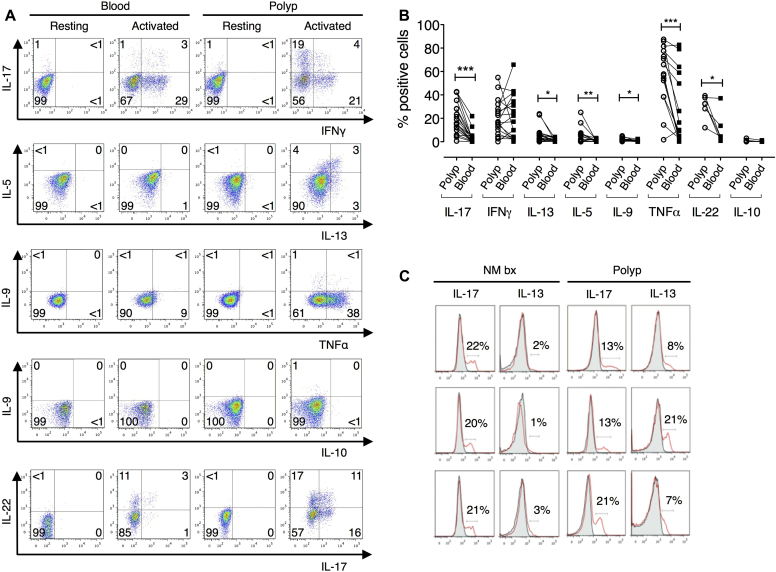
Differential expression of T_H_2/T_H_17 cytokines by polyp- and normal nasal mucosa–derived CD4^+^ cells. **A,** Representative staining on paired CD4^+^ blood and polyp cells. **B,** Percentages of polyp versus blood CD4^+^ cells producing cytokines (Wilcoxon matched-pairs signed-rank test, n = 6-18). **C,** IL-17 and IL-13 histograms for CD4^+^ biopsy and polyp cells (n = 3). Each *row* indicates an individual subject. *Gray*, Resting; *red*, activated. *NM bx*, Healthy nasal mucosa biopsy specimen. *P < .05, **P < .01, and ***P < .001.

Intracellular cytokine staining was performed on CD4^+^ T cells expanded from polyp explants and peripheral blood in parallel to establish the T_H_ cell cytokine profile. CD4^+^ T cells derived from polyps expressed significantly higher percentages of IL-17^+^ and IL-22^+^ cells together with T_H_2 cytokine (IL-5, IL-9, and IL-13)–producing cells (Fig 1, *A* and *B*), all of which showed negligible expression in expanded peripheral blood CD4^+^ T cells from the same donors. In addition, coexpression of IL-17 with IL-22 and IFN-γ was detected (see Fig E2 in this article's Online Repository at www.jacionline.org). A significantly higher percentage of polyp T cells produced the proinflammatory cytokine TNF-α, although IFN-γ expression was equivalent in CD4^+^ T cells from both sources.

### T_H_2 cytokine production is specific to CRSwNP, but T_H_17 cytokines are produced by nasal T cells from normal and inflamed tissue

**Fig E3:**
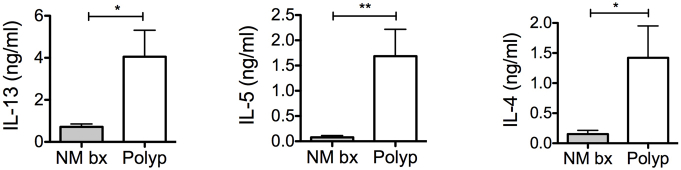


We next examined whether this cytokine expression profile in polyp explants was disease or tissue specific. Therefore T-cell phenotypes were compared with those from nasal mucosal biopsy specimens from healthy volunteers. IL-17 was produced by a comparable percentage of T cells derived from healthy nasal and nasal polyp explants (Fig 1, *C*) and confirmed at the protein level in cell-culture supernatants. Minimal IL-13^+^ cells were observed in the healthy nasal mucosa (Fig 1, *C*). Although IL-4 expression was not examined by using flow cytometry, significantly increased IL-4 levels, in addition to IL-5 and IL-13 levels, were detected in the supernatants of polyp explant cultures compared with those seen in healthy nasal mucosa explants (see Fig E3 in this article's Online Repository at www.jacionline.org).

### IL-17RB is expressed by *in vitro* T_H_2-polarized but not T_H_1-polarized cells

**Fig 2:**
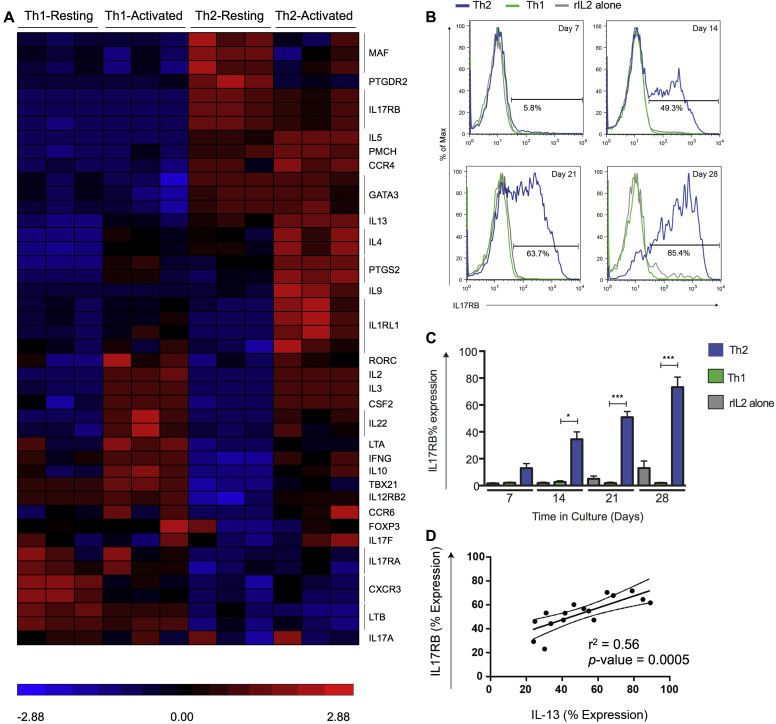
IL-17RB is a marker of T_H_2 cells. **A,** Comparison of activated T_H_1 versus T_H_2 samples identified 292 differentially expressed genes. The heat map shows selected T_H_1/T_H_2-associated genes. **B,** Representative data for IL-17RB expression by CD4^+^ cells cultured with IL-2/T_H_1/T_H_2 differentiation conditions. **C,** Mean frequency of IL-17RB^+^ cells in culture over time (T_H_1/T_H_2, n = 7-11; rIL-2 alone, n = 3-6). **D,** Linear regression analysis of IL-17RB/IL-13 expression in T_H_2 conditions (n = 4). *P < .05 and ***P < .001.

The IL-25 receptor IL-17RB is associated with T_H_2 cells and the promotion of T_H_2 responses.^9^, ^11^ We sought to examine IL-17RB expression in homogenous human T_H_1/T_H_2 CD4^+^ populations differentiated from naive peripheral blood T cells, as previously described.^25^ Differentiated cells were highly polarized toward a T_H_1 (IFN-γ^+^, T-box transcription factor [T-bet]^+^, and IL-12 receptor β2 [IL-12Rβ2]^+^) or T_H_2 (IL-4^+^, IL-5^+^, GATA-3^+^, and chemoattractant receptor-homologous molecule expressed on T_H_2 cells [CRTH2]^+^) phenotype, and a significant increase in *IL17RB* gene expression was observed in T_H_2 versus T_H_1 cell lines (Fig 2, *A*). IL-17RB expression increased with time in *in vitro* T_H_2-polarized T-cell cultures only (Fig 2, *B* and *C*), which followed similar kinetics to type 2 cytokine production (data not shown). Furthermore, IL-17RB expression was correlated with IL-13 expression in T_H_2 cell cultures (Fig 2, *D*). Together, these data suggest IL-17RB to be a robust marker of human T_H_2 cells.

### IL-17RB^+^ cells are a distinct T_H_2 cell population present in nasal polyps

**Fig E4:**
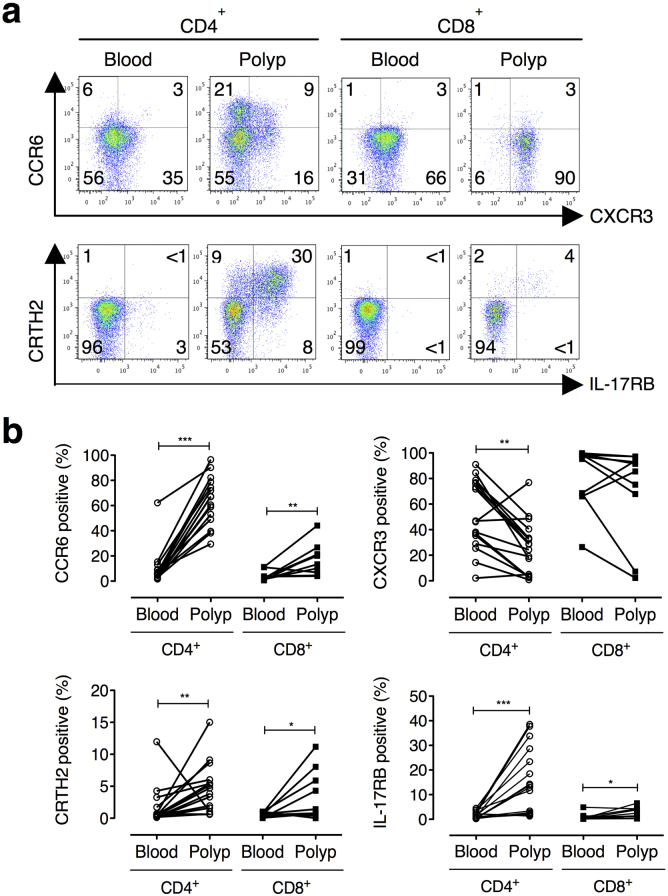


**Fig 3:**
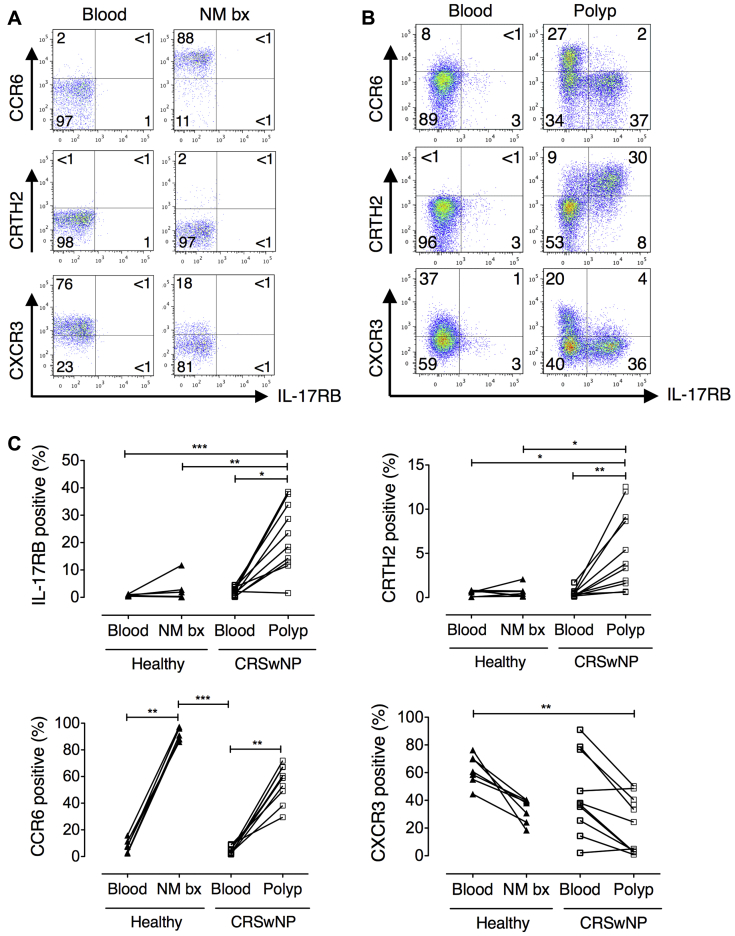
IL-17RB is expressed exclusively by polyp CD4^+^ T cells. **A** and **B,** Representative staining for T-cell phenotypic markers by polyp, healthy nasal biopsy, and paired peripheral blood cells. **C,** Expression of phenotypic markers by CD4^+^ T cells derived from blood and nasal tissue of healthy volunteers (n = 7) or patients with CRSwNP (n = 11; Kruskal-Wallis test with Dunn multiple comparison test). *P < .05, **P < .01, and ***P < .001.

We next examined whether T-cell expression of IL-17RB is also a feature of target organ tissue CD4^+^ cells in eosinophilic polyps. A substantial proportion of polyp CD4^+^ T cells expressed IL-17RB, whereas negligible IL-17RB expression was observed in matched peripheral blood or healthy nasal mucosal specimens (Fig 3). Coexpression of IL-17RB with the T_H_2-associated prostaglandin D_2_ receptor CRTH2 (Fig 3, *B*) was also detected, but IL-17RB expression was negligible on T_H_17-associated CCR6^+^ or T_H_1-associated CXCR3^+^ cells. Consistent with the high frequency of IL-17^+^ cells, an abundance of CCR6-expressing cells was also found in both healthy nasal mucosa and polyp explants (Fig 3, *A* and *C*). CD8^+^ cells showed similar surface molecule expression patterns to CD4^+^ cells, although lower percentages of cells positive for the surface molecules examined were generally observed (see Fig E4 in this article's Online Repository at www.jacionline.org).

**Fig E5:**
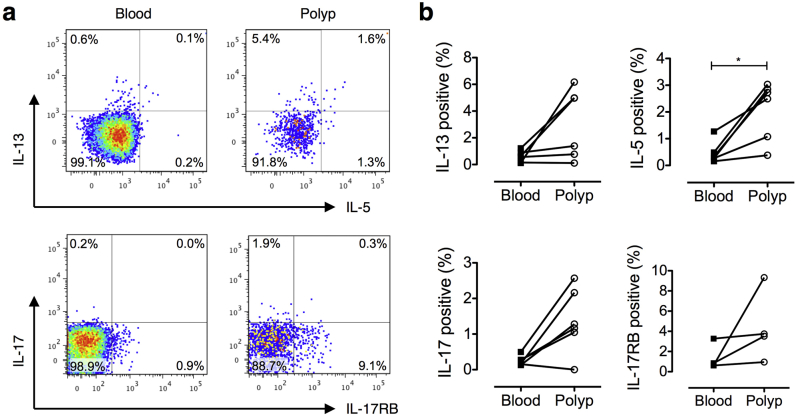


Although short-term cultures were used to generate sufficient cell numbers for experimentation, flow cytometric analysis of polyp tissue T cells immediately after collagenase digestion confirmed IL-17RB expression was not a culture artifact (see Fig E5 in this article's Online Repository at www.jacionline.org). Furthermore, percentages of T_H_2 and IL-17–producing cells were increased in digested polyp- versus blood-derived cells, which is consistent with findings from explant cultures.

### IL-17RB^+^CD4^+^ cells derived from nasal polyp explants represent *in vivo* differentiated memory T_H_2 cells

**Fig E6:**
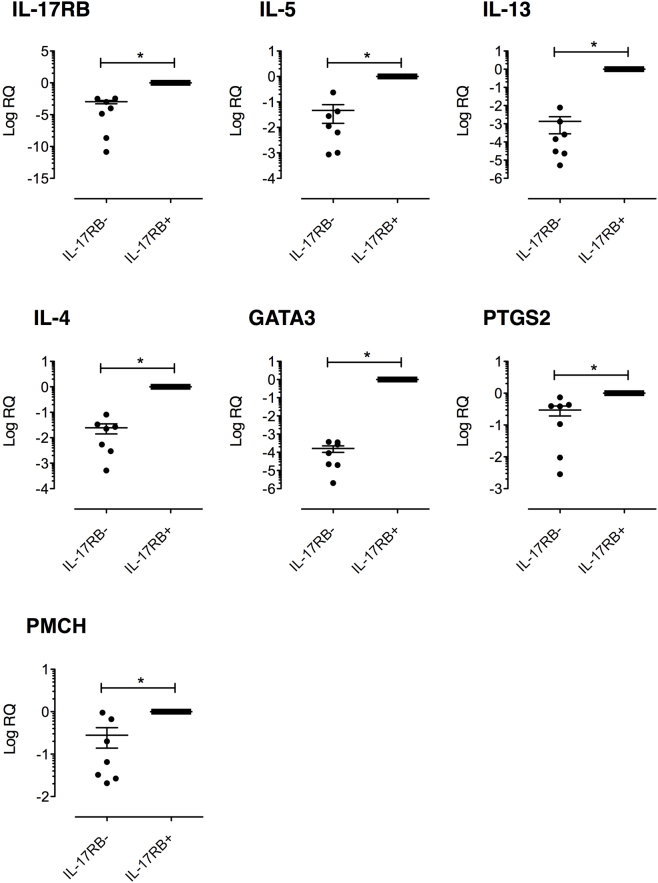


**Fig 4:**
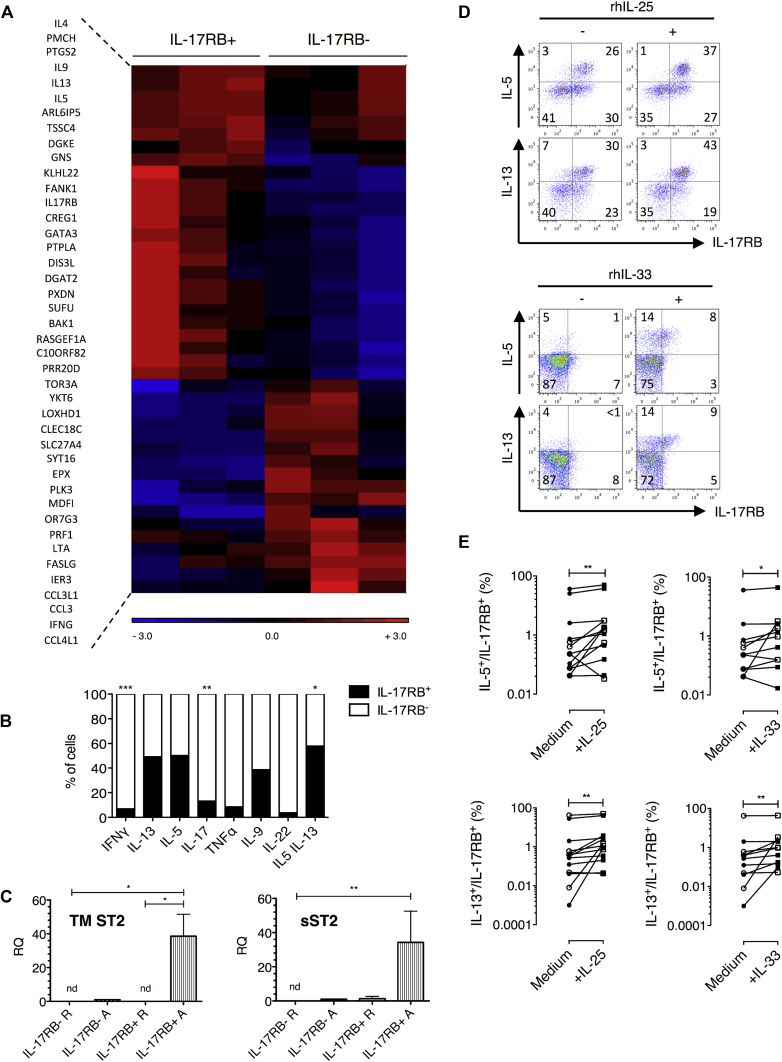
Polyp-derived CD4^+^IL-17RB^+^ cells have a T_H_2 profile and respond to IL-25 and IL-33. **A,** Heat map of 42 differentially expressed genes in polyp IL-17RB^+^ versus IL-17RB^−^ cells (n = 3). **B,** Cytokine-producing cells coexpressing IL-17RB (n = 5-13). **C,** Transmembrane and soluble ST2 mRNA expression (n = 4; Mann-Whitney test). **D,** Representative staining for polyp CD4^+^ cells with or without IL-25/IL-33. **E,** IL-5^+^/IL-13^+^ cells coexpressing IL-17RB with or without IL-25/IL-33. *Open symbols*, Day 0 addition (n = 5); *solid symbols*, day 7 addition (n = 8). The Wilcoxon test was used. *P < .05, **P < .01, and ***P < .001.

To further address the phenotype of IL-17RB^+^CD4^+^ cells from nasal polyp explants, explant-derived cells were sorted by means of fluorescence-activated cell sorting for IL-17RB^+^CD4^+^ expression after short-term expansion. IL-17RB^−^CD4^+^ cells were also sorted for comparison. T_H_2-associated genes, including *IL4*, *IL5*, *IL9*, *IL13*, and *GATA3*, showed considerable upregulation in activated IL-17RB^+^CD4^+^ versus activated IL-17RB^−^CD4^+^ cells (Fig 4, *A*), with differential expression for a majority of these genes reaching statistical significance (see Table E3 in this article's Online Repository at www.jacionline.org). Furthermore, correspondingly lower expression of T_H_1-associated genes, including *IFNG*, *LTA*, and *CCL3*, was identified. Moreover, the genes for promelanin-concentrating hormone and prostaglandin-endoperoxide synthase 2 were preferentially expressed in IL-17RB^+^ cells in line with data from *in vitro* polarized T_H_2 cultures (Fig 2, *A*) and previously published findings.^26^, ^27^ Microarray-based gene expression results were confirmed by using quantitative RT-PCR analysis (see Fig E6 in this article's Online Repository at www.jacionline.org).

### IL-17RB^+^ cells predominantly and selectively produce T_H_2 cytokines

We next examined whether IL-17RB expression colocalized with T_H_2 cytokines in nasal polyp explant T-cell cultures. Fig 4, *B*, shows the percentage of cells expressing IL-17RB when segregated by cytokine production. IL-5-producing, IL-13-producing, and IL-5/IL-13–coproducing cells were approximately 5 times more likely to coexpress IL-17RB compared with T_H_1/T_H_17 cytokine–producing cells (ie, 52% of IL-5–producing cells were IL-17RB^+^, whereas 8% of IFN-γ–producing cells were IL-17RB^+^). In addition, IL-17RB^+^ cells were accountable for the majority of IL-5/IL-13–coproducing T cells (59%; Fig 4, *B*). Notably, percentages of IFN-γ–and IL-17–producing cells were significantly lower in the IL-17RB^+^ population compared with those in the IL-17RB^−^ population. A similar trend was observed for TNF-α and IL-22.

### The IL-33 receptor ST2 is also expressed by IL-17RB^+^ cells

T cells from nasal polyp explants were next examined for mRNA expression of the IL-33 receptor ST2. Expression of transmembrane and soluble isoforms (sST2) of *ST2*, as measured by using quantitative RT-PCR, were increased in activated IL-17RB^+^ cells compared with IL-17RB^−^ cells (Fig 4, *C*), suggesting that IL-17RB^+^ T cells might also be IL-33 responsive.

### IL-17RB and ST2 are functional and potentiate T_H_2 cytokine production by nasal polyp T cells

T_H_2 cytokine expression was determined by means of flow cytometry in polyp explants cultured in the presence of recombinant human IL-25 or IL-33 to evaluate whether IL-17RB and ST2 expressed on polyp T cells were functional (Fig 4, *D*). Recombinant cytokines were added either on the day of explantation or day 7 after stimulation. Analysis was performed 7 days later. Addition of IL-25 induced a mean 1.5-fold increase in the percentage of IL-17RB^+^IL-5^+^CD4^+^ T cells and a 1.4-fold increase in the percentage of IL-17RB^+^IL-13^+^CD4^+^ T cells in explant cultures (Fig 4, *E*). Addition of IL-33 had a comparable effect to IL-25, with a mean 1.4-fold increase in the percentage of IL-17RB^+^IL-5^+^CD4^+^ T cells and a 1.2-fold increase in the percentage of IL-17RB^+^IL-13^+^CD4^+^ T cells. Time of recombinant cytokine addition had no effect on the response of IL-17RB^+^ST2^+^ cells. Addition of IL-25 to polyp-derived T cells at day 7 after stimulation was still associated with a significant increase in IL-17RB^+^IL-5^+^ and IL-17RB^+^IL-13^+^ CD4^+^ T-cell counts (data not shown).

### Nasal polyp epithelium and eosinophils express IL-25

**Fig E7:**
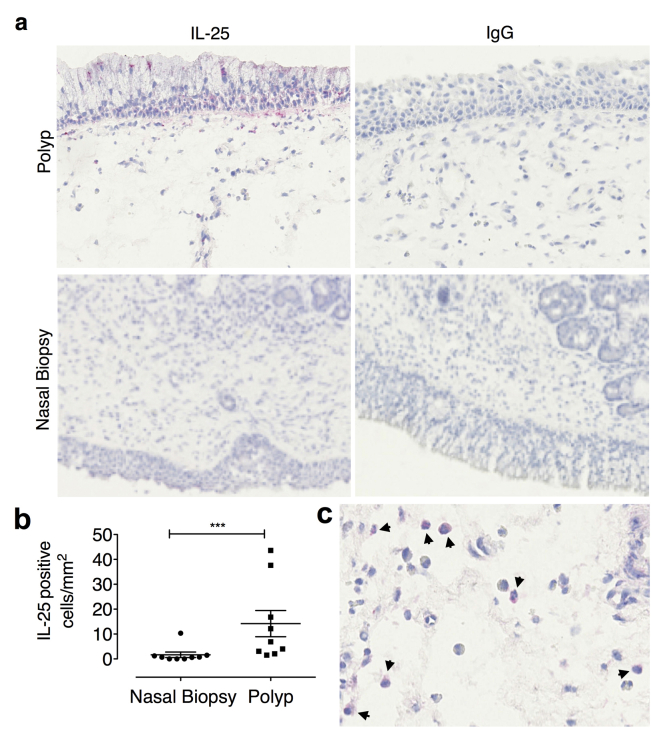


**Fig E8:**
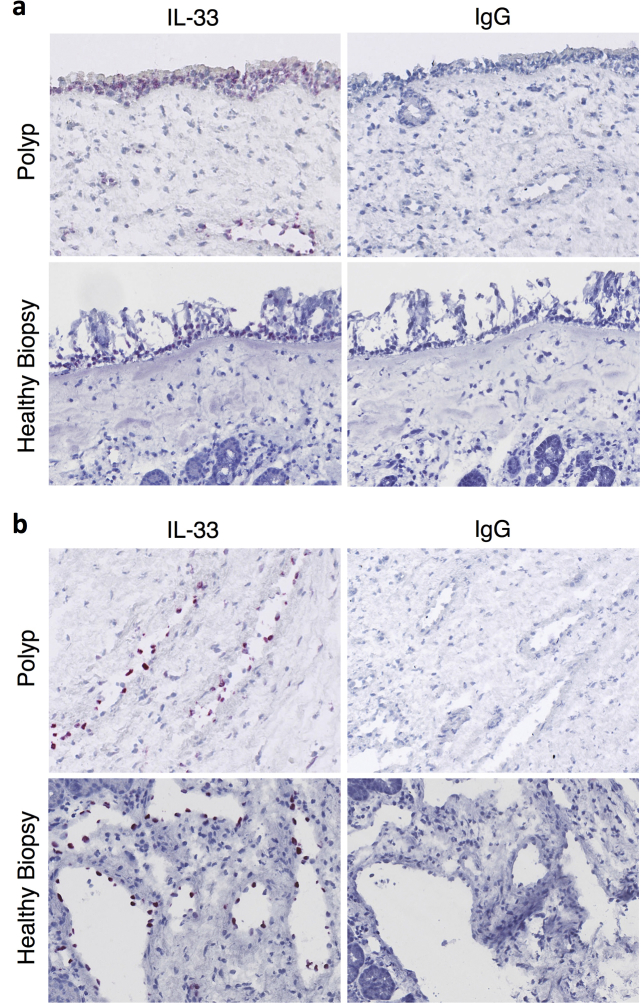


Cellular sources of IL-25 within nasal polyp tissue were investigated by using immunohistochemistry. Immunostaining was observed in the epithelium of nasal polyps but not in healthy control biopsy tissue (see Fig E7, *A*, in this article's Online Repository at www.jacionline.org). Furthermore, a significantly higher number of IL-25^+^ cells were present in the polyp submucosa (see Fig E7, *B*). These cells were identified to be eosinophils based on cell morphology (see Fig E7, *C*). In contrast, immunoreactive IL-33 was detected in both nasal polyp and healthy biopsy tissue, with immunostaining indicating a predominantly epithelial and endothelial pattern of expression (see Fig E8 in this article's Online Repository at www.jacionline.org).

### IL-17RB^+^ and IL-17RB^−^ cells have distinct T-cell receptor specificities with common T-cell receptor clones exhibited by IL-17RB^+^ cells

We next examined whether nasal IL-17RB^+^CD4^+^ T_H_2 cells in patients with CRSwNP represent oligoclonal populations driven by *in vivo* antigen or superantigen expansion. Clonality was examined by T-cell receptor variable β-chain (TCR Vβ) analysis with the immunoSEQ assay and compared in IL-17RB^+^CD4^+^ and IL-17RB^−^CD4^+^ cells sorted from nasal polyp explant cultures of 4 patients with CRSwNP. No skewing of TCR Vβ family use was observed (data not shown), but sequencing of complementarity-determining region 3 (CDR3) regions revealed that polyp IL-17RB^+^CD4^+^ cells contained a smaller number of unique clones compared with IL-17RB^−^CD4^+^ cells in all 4 cases analyzed (Table I). Additionally, less than 1% of sequenced clones were present within both IL-17RB^+^CD4^+^ and IL-17RB^−^CD4^+^ populations. Remarkably, 2 distinct common clones in IL-17RB^+^CD4^+^ T cells, identified to belong to the Vβ5.2 and Vβ6 families by using immunoSEQ analysis, were present in 3 of 4 patients with CRSwNP studied. Overall, these results suggest that polyp IL-17RB^+^CD4^+^ T cells have undergone clonal expansion and that common epitopes might drive this process, even in different patients.

### T_H_17 cells are the default T_H_ cell phenotype in normal nasal mucosal immunity

**Fig 5:**
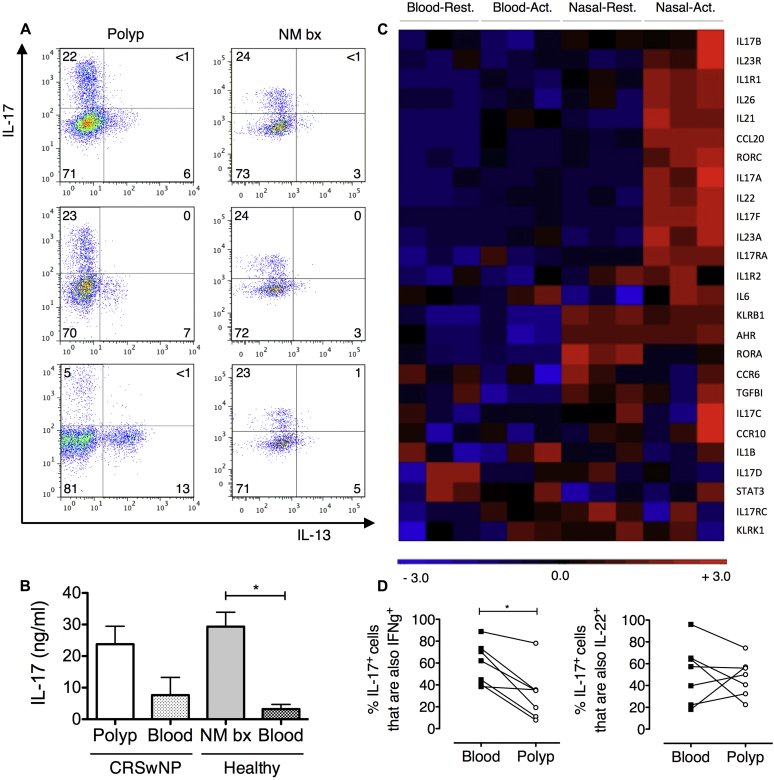
A T_H_17 signature characterizes CD4^+^ T cells of the healthy nasal mucosa. **A,** Representative IL-13/IL-17 staining in polyp and healthy nasal mucosa biopsy specimen *(NM bx)* CD4^+^ cells (n = 3). **B,** IL-17 expression in explant culture supernatants (n = 7, mean + SEM; Mann-Whitney test. **C,** Heat map of T_H_17 genes in NM bx versus blood CD4^+^ cells (n = 3). **D,** IL-17 coexpression with IFN-γ/IL-22 in blood versus polyp CD4^+^ cells (n = 6; Wilcoxon matched-pairs signed-rank test). *P < .05.

Given the abundant expression of IL-17 by CD4^+^ T cells derived from the healthy nasal mucosa in addition to nasal polyps, these cells were characterized further. In agreement with CCR6 and IL-17RB expression data (Fig 3), no coexpression of IL-17 and IL-13 was detected (Fig 5, *A*). In supernatants of CD3/CD28-stimulated T cells, IL-17 was produced by T cells derived from both healthy nasal mucosa and polyp tissue but not peripheral blood–derived T cells from the same patients (Fig 5, *B*).

**Fig E9:**
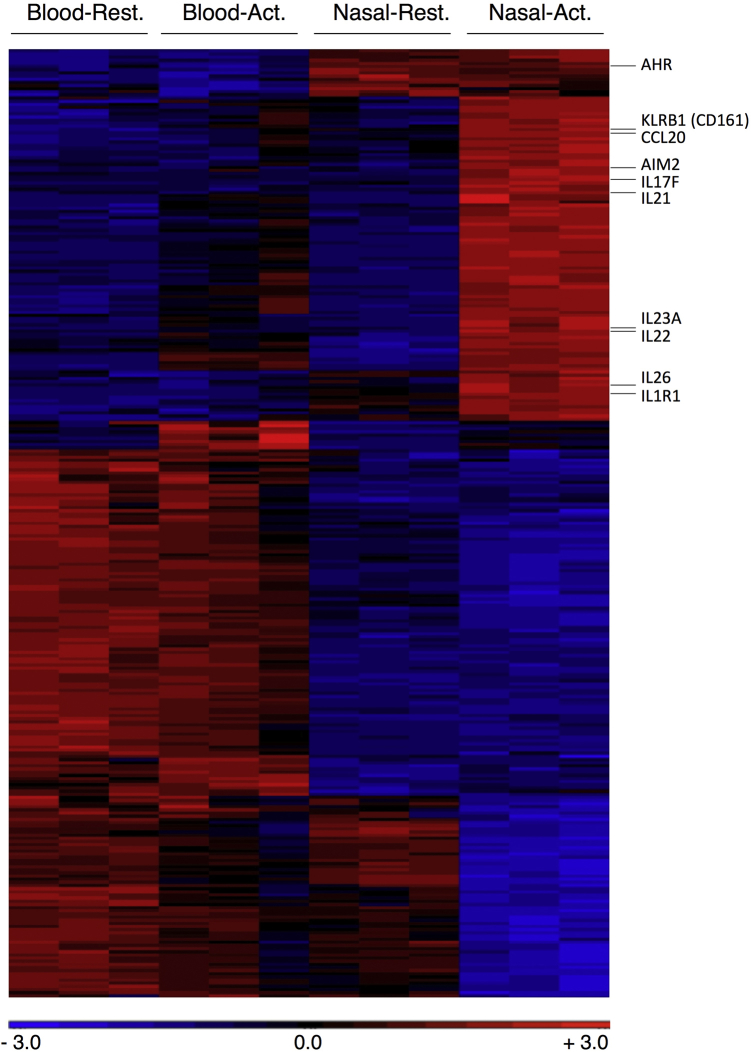


CD4^+^ T-cell populations were also sorted from paired nasal explant and peripheral blood cultures for transcriptome profiling (see Fig E9 in this article's Online Repository at www.jacionline.org). Preferential expression of T_H_17-associated genes was observed in activated nasal CD4^+^ cells. Of note, the 5 genes that were most highly overexpressed in nasal versus peripheral blood CD4^+^ T cells were all T_H_17 associated: *IL17F*, *IL22*, *CCL20*, *KLRB1* (CD161), and *IL1R1* (see Table E4 in this article's Online Repository at www.jacionline.org). Significant overexpression of the gene for the DNA-sensing inflammasome component absent in melanoma 2 *(AIM2)* was also observed in nasal mucosal T cells. Analysis of additional selected T_H_17-associated genes further revealed preferential expression of *IL17A*, *IL21*, *IL23*, *IL23R*, aryl hydrocarbon receptor *(AHR)*, and *RORC* (Fig 5, *C*) by activated nasal CD4^+^ cells. These data suggest that the healthy, homeostatic T-cell response in the nasal mucosa is associated with a strong T_H_17 signature compared with the periphery.

### T_H_17 cells in nasal polyps have a potentially protective immune homeostatic role associated with reduced IFN-γ coexpression

T_H_17 cells can coproduce IFN-γ and IL-22. IL-17/IFN-γ double-positive cells have been associated with a pathogenic proinflammatory phenotype, whereas IL-17/IL-22 double-positive cells have been reported to have protective properties by inducing expression of antimicrobial peptides.^28^, ^29^, ^30^ Lower coexpression of IFN-γ by IL-17^+^ T cells from polyp explants was found compared with that seen in blood-derived cells (Fig 5, *D*). No difference was observed in the percentages of IL-17^+^ cells coexpressing IL-22.

## Discussion

Recently, ILC2s have been identified in nasal polyps,^18^, ^19^, ^31^ and the presence of T_H_2 cells in white patients with CRSwNP has been demonstrated.^32^ However, the local T-cell response itself remains relatively uncharacterized. Here, using a short-term explant model to expand and study T cells from surgical specimens, we report a significant population of IL-17RB–expressing T_H_2 cells in nasal polyps with a gene expression profile akin to that of highly polarized T_H_2 cells.^25^, ^26^ Approximately 50% of IL-5^+^IL-13^+^ polyp-derived CD4^+^ T cells expressed IL-17RB, suggesting IL-17RB^+^ cells represent a subset of T_H_2 cells.

We demonstrate that IL-17RB^+^CD4^+^ cells from polyps express mRNA for both transmembrane and soluble isoforms of ST2 on activation and respond to both IL-25 and IL-33 with augmented IL-5 and IL-13 production. ST2 expression by *in vitro* differentiated human peripheral blood T_H_2 cells has been described,^33^ and both IL-25 and IL-33 receptors are expressed and functional on human and murine ILC2s.^14^, ^18^, ^19^, ^34^ However, the role of these pathways in human mucosal T-cell responses has not been examined. These data now establish a direct link of IL-25, IL-33, and T_H_2 cells in human disease and suggest that IL-17RB^+^ST2^+^ T_H_2 cells likely contribute to CRSwNP pathogenesis through the IL-25/IL-33 axis. We found increased IL-25 immunostaining in polyps, localizing to eosinophils and epithelial cells, which is consistent with previously published reports^11^, ^12^, ^13^ and in agreement with the increased IL-25 mRNA expression seen in patients with eosinophilic CRSwNP reported by Iinuma et al.^35^ In addition, constitutive expression of IL-33 was detected in epithelium and endothelium of both healthy and polyp nasal tissue, which is in line with mRNA expression studies.^31^, ^36^, ^37^ These findings suggest that these cells might be endogenous sources of IL-25 and IL-33 in nasal polyps. However, the mechanism of IL-33 release is yet to be elucidated.

Colonization with *S aureus* in nasal polyposis is associated with high levels of IgE,^38^ and *S aureus* superantigens, such as staphylococcal enterotoxin B, can drive the T_H_2-type response in eosinophilic polyps.^5^, ^39^ Here we demonstrate that nonrandom segregation of unique CDR3 clones occurs with 2 CDR3 clones present in the IL-17RB^+^ population in 3 of 4 samples analyzed. Although these results require confirmation in a larger study, they are suggestive of oligoclonality in the TCR Vβ repertoire within the IL-17RB^+^ polyp T-cell population and indicate possible expansion by common antigens in different patients. Routine skin prick testing in these patients with CRSwNP did not identify coincidental sensitization to a common aeroallergen (data not shown). Furthermore, the Vβ5.2 and Vβ6 families are reported to be preferentially expressed by cutaneous lymphocyte–associated antigen–positive cells responding to the superantigen staphylococcal enterotoxin A in patients with atopic dermatitis and induced by the toxic shock syndrome toxin 1 superantigen, respectively.^40^, ^41^ Although speculative, this raises the possibility that local IL-17RB^+^ T_H_2 cells in patients with CRSwNP undergo antigen-specific expansion in response to common but as yet undefined epitopes with an additional non–antigen-specific component mediated by superantigens.

We demonstrate that the T_H_ response in the healthy nasal mucosa is heavily biased toward T_H_17 responses compared with the periphery. Although we did not examine the relative dominance of the T_H_17 phenotype compared with other T_H_ cell phenotypes, we observed that the 5 most overexpressed genes in normal nasal mucosal T cells compared with peripheral blood T cells were all strongly T_H_17 associated. We propose that a significant population of nasal T cells differentiate into T_H_17 cells *in vivo*, with the propensity to produce IL-17 and related cytokines should they become activated *in vivo*.^42^ We hypothesize that this T_H_17 phenotype represents a key part of the nasal mucosal host defense response. Priming of autologous monocytes with pathogens, such as *S aureus* and *Candida albicans*, induces T_H_17 responses in naive human T cells,^43^ suggesting that chronic exposure of the nasal mucosa to nonpathogenic and pathogenic microorganisms, such as *Staphylococcus epidermidis*, *S aureus*, and corynebacteria, could be the mechanism behind this response.

Within the T cells derived from healthy nasal tissue, we found that transcripts encoding IL-17F and IL-22 were the most highly upregulated. IL-17A and IL-17F are homologous molecules sharing 55% amino acid identity.^44^ Both induce expression of numerous chemokines, cytokines, and adhesion molecules, although IL-17A is more effective at inducing inflammatory gene expression.^28^, ^45^, ^46^, ^47^ IL-17F is expressed by a wide variety of tissue, including in the lung,^47^, ^48^ and can also potentiate IL-22–induced expression of antimicrobial peptides.^28^ Thus the presence of T cells able to produce IL-17F and IL-22 is suggestive of a function for these cells in nasal mucosal immune homeostasis. Microarray analysis also identified overexpression of AIM2 mRNA in nasal explant CD4^+^ T cells. The AIM2 inflammasome is activated by intracellular pathogens, leading to caspase-1–dependent IL-1β secretion.^49^, ^50^ Further studies will be needed to examine whether this innate pathway is functional in nasal T_H_17 cells.

Our study has some limitations. For example, memory T cells were phenotyped after short-term expansion. Therefore it is possible that a proportion of CD45RA^+^ peripheral blood T cells acquired CD45RO expression during culture and might have retained some of their baseline CD62 ligand and CCR7 expression characteristics. In addition, IL-17RB–expressing T cells were mainly characterized after *in vitro* expansion. Analysis of freshly isolated IL-17RB^+^ T cells from digested polyps was hampered by low cell numbers and lower IL-17RB expression, possibly reflecting the effects of enzymatic digestion, and therefore data were obtained from fewer cases. The IL-17RB mAb used in these studies did not prove suitable for immunohistochemical analysis, and further studies will be needed for *in vivo* expression analysis of IL-17RB. Finally, the effect of IL-25 and IL-33 stimulation on T_H_2 responses *in vitro* was modest, although the concentrations of recombinant IL-25 and IL-33 used in this study were similar to previously published reports.^12^, ^35^

Nonetheless, our data establish a biological link between IL-17RB expression and responsiveness to IL-25 in T_H_2 cells derived from polyps. Further optimized culture studies will be needed to characterize this response fully. Although 2 recent studies have reported the existence of IL-17RB^+^ cells in patients with CRSwNP,^35^, ^51^ our findings represent the first direct colocalization of IL-17RB with T_H_2 cells.^35^

In conclusion, we identify functional IL-17RB as a marker of local T_H_2 cells present in chronically inflamed nasal polyp tissue from patients with CRSwNP. Coexpression of ST2 by these cells, in addition to ILC2s, indicates that the IL-25/IL-17RB and IL-33/ST2 pathways could be attractive therapeutic targets. In addition, these data also provide novel insights into mechanisms of nasal immune homeostasis and suggest a role for T_H_17 cells in this process.

**Key messages**

• For the first time, we show that local IL-17RB^+^ T_H_2 cells in nasal polyps coexpress ST2 and that both receptors function, in response to their respective ligands IL-25 and IL-33, to potentiate T_H_2 cytokine production.
• IL-17RB^+^ T_H_2 cells express common TCR clones, which is suggestive of recognition, clonal expansion, or both of T cells driven by a common antigen or antigens in patients with CRSwNP.
• T_H_17 cells are present in the nasal mucosa as part of the normal homeostatic immune response.

## Figures and Tables

**Table I:** TCR Vβ repertoire analysis of IL-17RB^+/−^ cells

Patient ID	HKP020		HKP023		HKP026		HKP036	
Cell population	IL-17RB^+^	IL-17RB^−^	IL-17RB^+^	IL-17RB^−^	IL-17RB^+^	IL-17RB^−^	IL-17RB^+^	IL-17RB^−^
Total clones (productive)	4,871	1,146	969	3,801	1,896	443,183	2,435	47,486
Unique clones (no.)	33	91	28	97	55	6,475	113	1,759
Shared clones	0		1		25		11	
Common clones								
CASSLNTGYEQYF	+	−	+	+	+	−	−	−
CASSYPGEAFF	+	−	+	−	−	−	+	−

Numbers of unique TCR clones present in sorted polyp-derived CD4^+^IL-17RB^+^ and CD4^+^IL-17RB^−^ populations analyzed by using the immunoSEQ assay are shown (n = 4 separate donors). Amino acid sequences represent CDR3 regions of 2 common clones identified within the IL-17RB^+^ population of at least 3 of the 4 donors.

## Supplementary data

Online Repository Data
