# Supplementary material for: IL-25/IL-33–responsive TH2 cells characterize nasal polyps with a default TH17 signature in nasal mucosa
Source: J Allergy Clin Immunol. 2016 May;137(5):1514–24. doi: 10.1016/j.jaci.2015.10.019 (PMC4852988; doi:10.1016/j.jaci.2015.10.019)
Supplement: Online Repository Data [file mmc1.docx]

**MATERIALS AND METHODS**

**Subjects**

Peripheral blood and nasal polyp tissues were collected from CRSwNP patients during polypectomy surgery. Peripheral blood and normal nasal mucosal biopsies from the inferior turbinate were collected from healthy volunteers. Demographic data for CRSwNP patients and healthy volunteers are shown in Table 1. For Th1/Th2 differentiation cultures, peripheral blood was obtained from atopic and non-atopic donors. All subjects provided written, informed consent and all studies were approved by the Research Ethics Committees of Guy’s Hospital, Royal Brompton Hospital and Royal National Throat Nose Ear Hospital, London. Collected tissue was treated in accordance with the Human Tissue Act 2004.

**PBMC cultures**

Peripheral blood mononuclear cells (PBMCs) were isolated from peripheral blood using Lymphoprep (Axis-Shield, Dundee, UK) according to the manufacturer’s instructions. PBMCs were resuspended in RPMI-1640 medium supplemented with 10% fetal bovine serum (Life Technologies, Paisley, UK), 100 U/ml penicillin, 100 μg/ml streptomycin and 2 mM L-glutamine (Life Technologies) – hereafter referred to as complete medium. PBMCs were plated at 1 x 10^6^ cells/ml, supplemented with rIL- 2 (50 U/ml; Novartis, Camberley, UK) and cultured at 37°C, 5% CO2 for 2-3 days. Cells were then stimulated by seeding onto tissue culture plates pre-coated with anti- CD3 antibody (1 mg/ml; OKT3, ECACC) and anti-CD28 antibody (1 mg/ml; 15E8; Sanquin, Amsterdam, Netherlands). Cells were cultured for a further 7 days before analysis with removal from stimulation after 3-4 days. Fresh complete medium and IL-2 were added every 2-3 days or when required. In some experiments, rhIL-25 (125 ng/ml; R&D Systems, Abingdon, UK) and rhIL-33 (50 ng/ml; R&D Systems) were added to samples on day 0 of culture or on day 7 post-stimulation.

**Th1/Th2 differentiation**

Th1/Th2 *in vitro* differentiated cultures were performed as previously described.^E1^ Briefly, CD4+ T cells were isolated from PBMCs using a CD4 Positive Isolation Kit (Dynabeads, Life Technologies) according to the manufacturer’s instructions. Purified naïve CD45RA+ cells were obtained by depletion of CD45RO+ cells with mouse anti- human CD45RO antibody (UCHL1; BD Biosciences, Oxford, UK) and rat anti-mouse IgG Dynabeads (Life Technologies) according to the manufacturer’s instructions. Purified CD45RA+ cells were then stimulated with anti-CD3/CD28 and cultured at 1 × 10^6^ cells/ml in complete medium with the addition of rIL-2 and polarizing cytokines as required.

For Th1 differentiation, rIL-12 (2.5 ng/ml; R&D Systems), anti-IL-4 (5 μg/ml; 7A3-3; ECACC) and anti-IL-10 (5 μg/ml; JES-19F1; ATCC) were added to cultures. For Th2 differentiation, rIL-4 (12.5 ng/ml; NBS Biologicals, Huntingdon, UK), anti-IFNγ (5 μg/ml; 7R2/A4; ECACC), and anti-IL-10 (5 μg/ml; JES-19F1) were added. Cells were restimulated with anti-CD3/28 antibodies every 7 days and cultured for up to 28 days to obtain highly polarized Th1/Th2 cells.

**Tissue explant cultures**

Nasal polyp and normal nasal mucosa tissues were dissected and resuspended in complete medium. Tissues were cultured at 37°C, 5% CO_2_ in the presence of rIL-2 (50 U/ml). After 2-3 days, cells were passed through a 0.2 μm cell strainer to obtain single cell suspensions and restimulated by culturing on anti-CD3/28 antibody coated plates. Cells were cultured for a further 7 days before analysis with removal from stimulation after 3-4 days. Blood and parallel tissue samples were analyzed on the same day. For blood cultures with rhIL-25 (125 ng/ml; R&D Systems) and rhIL-33 (50 ng/ml; R&D Systems) added from day 0 or on day 7 post-stimulation, the analogous polyp tissue culture was also subjected to the same treatment.

**Collagenase tissue digestion**

Nasal polyp tissue was placed in a petri dish containing 3 mM CaCl_2_/HBSS for mechanical dissociation. Tissue was subsequently transferred to a 24 well plate and 100 μl Liberase (thermolysin low) collagenase (2.5mg/ml; Roche, Burgess Hill, UK) was added to wells to a final volume of 2 ml/well. The plate was wrapped in foil and placed on a rocking platform overnight at 4°C. The next day, dissociated cells were passed through a 100 μM cell strainer and washed before resuspension in complete RPMI-1640. Cells were then counted and analyzed by flow cytometry.

**Flow Cytometry**

**Antibodies:** Anti-human antibodies used in this study were obtained from the following companies: BD Biosciences – CCR6-PE/Cyanine (Cy) 7 (G034E3), CD8- Brilliant Violet^TM^ (BV) 510 (RPA-T8), IL-17A-BV421 (N49-653) and IL-5-PE (TRFK5); Biolegend, Cambridge, UK – CD4-Peridinin chlorophyll (PerCP)/Cy5.5 (OKT4), CCR6-FITC (G034E3), CRTH2-PE (BM16), CXCR3-BV421 (G025H7), IL-13-PE (JES10-SA2), IL-13-PerCP/Cy5.5 (JES10-SA2) and IL-17A-Alexa Fluor® (AF) 647 (BL168); eBioscience, Hatfield, UK – CD3-eFluor450 (OKT3), CD4-FITC (RPA-T4), CD4-PE/Cy7 (RPA-T4), IL-9-AF647 (MH9A4), IL-9-PE (MH9D1), IL-10-AF647 (JES3-9D7) and TNFα-FITC (MAb11); R&D Systems – IL-22-PE (142928). Anti- human IL-17RB antibody (clone D9.2)^E2^ was labeled in house with an AF647 antibody labeling kit (Life Technologies). Anti-human IFNγ antibody was produced in house from ECACC hybridoma 7R2/A4 and was labeled in house with an AF488 antibody labeling kit (Life Technologies).

**Surface staining:** Cells were washed with FACSFlow (BD Biosciences) and stained with viability dye eFluor780 (eBioscience) to exclude dead cells. Fc receptor blocking reagent (Miltenyi Biotec, Surrey, UK) was then added to block non-specific Fc receptor – mediated binding prior to addition of antibodies. Cells were incubated on ice (except CRTH2 staining, performed at room temperature) for 30 minutes before analysis on a BD FACSCanto II cytometer (BD Biosciences). Data was analyzed with FlowJo v7.6 software (Tree Star, Inc.).

**Intracellular cytokine staining:** Cells were activated by incubation with PMA (5 ng/ml) and ionomycin (500 ng/ml) for 4 hours at 37°C with monensin (2 μl/ml) added for the final 2 hours as previously described.^E1^ Cells with no PMA and ionomycin added were used as resting controls. Cells were stained with eFluor780 viability dye and for cell surface markers as above. Cells were then washed, fixed and permeabilized with Cytofix/Cytoperm and Perm/Wash (BD Biosciences) as per the manufacturer’s instructions prior to addition of intracellular cytokine antibodies. Analysis was performed on a BD FACSCanto II cytometer with FlowJo v7.6 software.

**Fluorescence-activated cell sorting:** Cells for sorting were stained as above and sorted by the Biomedical Research Centre (BRC) Flow Core Facility at Guy’s and St Thomas’ NHS Trust with the FACSAria II (BD Biosciences) flow cytometer. Purity of sorted cells was determined to be >95%.

**Immunohistochemistry**

Nasal polyp and nasal mucosal tissues were fixed in 4% paraformaldehyde (PFA) and embedded in O.C.T. compound before cryosectioning into 6μM sections. Sections were stained with 1:200 mouse anti-human IL-25 monoclonal antibody (182203; R&D Systems) or 1:100 mouse anti-human IL-33 antibody (Nessy-1; Enzo Life Sciences, Exeter, UK). Briefly, slides were washed and endogenous biotin and avidin binding sites blocked with the avidin/biotin blocking kit (Vector, Peterborough, UK). Slides were then blocked with 3% horse serum before incubation of primary antibody to sections at 4°C overnight in a humidified chamber. Mouse IgG was applied as the control for non-specific staining. Biotinylated horse anti-mouse IgG (1:100) was used as the secondary antibody with Vectorstain ABC-AP mouse IgG reagent (Vector) added to enhance sensitivity. Fast Red substrate (Sigma-Aldrich, Poole, UK) and Harris’s hematoxylin was used to develop and counterstain sections before mounting with Faramount medium (Dako, Ely, UK).

Whole slide imaging was performed with a Hamamatsu NanoZoomer (Welwyn Garden City, UK). Alternatively, staining was observed with an Olympus BX40 light microscope (Hamburg, Germany) and images captured with a JVC KY-F55B camera (London, UK) with Zeiss KS300 software (Cambridge, UK).

**Gene expression analysis**

**Microarray:** For the *in vitro* Th1/Th2 polarized microarray, all steps were performed as previously described.^E3^ For the polyp-derived IL-17RB+/- and blood/nasal CD4+ T cell arrays, cells were either incubated in the presence (activated cells) or absence (resting cells) of PMA/ionomycin for 4 hours prior to RNA isolation and microarray analysis. RNA was isolated from cell pellets using the miRNeasy mini kit and RNeasy MinElute cleanup kit (Qiagen, Manchester, UK) according to the manufacturer’s instructions. cDNA synthesis and amplification was performed with the Ovation PicoSL WTA system V2 kit (NuGEN, Leek, Netherlands) as per the manufacturer’s instructions. Purity and yield was then analyzed using the Bioanalyzer platform (Agilent, Stockport, UK) and NanoDrop 2000 spectrophotometer (Thermo Scientific, Loughborough, UK) respectively before amplified cDNA was biotin-labeled with the NuGEN Encore BiotinIL module according to the manufacturer’s instructions.

Biotin-labeled cDNA was hybridized at 48°C to an Illumina Human HT-12 v4 Expression BeadChip by the BRC Genomics Facility at Guy’s and St Thomas’ NHS Trust before scanning in the iScan system (Illumina, Essex, UK). Initial data analysis and quality control was performed using GenomeStudio^TM^ software (Illumina). All samples passed Illumina standard quality control procedures within GenomeStudio Gene Expression Module. Data was normalized using quantile normalization with no background correction in GenomeStudio. Gene level data was then exported from GenomeStudio using the Partek Report Plug-in, which performs log2 transformation of the data. Further data analyses were performed with Partek Genomics Suite^TM^ software (Partek Incorporated, Missouri, USA) using a 3 way-ANOVA model (Donor, cell type, activation) using standard gene expression workflows. The data has been deposited in the Gene Expression Omnibus (Accession numbers: GSE70898 and GSE70900).

**Quantitative real-time PCR (qRT-PCR):** TaqMan primer/probe sets used to perform qRT-PCR were obtained from Life Technologies: *IL1RL1* (full length transcript) Hs00249389_m1, *IL1RL1* (soluble transcript) Hs01073300_m1. 18s ribosomal RNA was used as the reference gene and analysis was performed on the ViiA^TM^ 7 Real Time PCR System qPCR machine utilizing the ViiA^TM^ 7 software (Life Technologies). Data was normalized to 18s rRNA expression (endogenous gene) and analyzed utilizing the comparative 2^–ΔΔCt^ method.

**T cell receptor variable beta chain (TCR Vβ) repertoire analysis**

DNA was isolated from cells using TRIzol (Life Technologies) as per the manufacturer’s instructions. Clean up was performed using the MinElute cleanup kit (Qiagen) according to the manufacturer’s instructions. TCR Vβ repertoire analysis of DNA samples was performed utilizing the immunoSEQ assay (Adaptive Biotechnologies, Seattle, USA). Data acquired was analyzed with the immunoSEQ analyzer software (Adaptive Biotechnologies).

**Cytometric bead array**

T cells expanded from tissue explants for 7 days were restimulated for 24 hours with anti-CD3/28 antibodies before harvesting of supernatants. Cytometric bead array (CBA) assays were performed on cell culture supernatants with the use of CBA Flex Sets (BD Biosciences) according to the manufacturer’s instructions. Samples were analyzed on the LSR Fortessa flow cytometer (BD Biosciences) with a high throughput sampler plate reader platform. Data was analyzed utilizing FlowJo^TM^ v7.6 software and GraphPad Prism® 5 software (GraphPad Software, California, USA).

**Statistical analysis**

Statistical analysis was performed using GraphPad Prism® 5 software. Results were considered statistically significant if *p<0.05.* Significance was indicated as *p<0.05*, p<0.01*** and *p<0.001***.*

**REFERENCES**

1. Cousins DJ, Lee TH, Staynov DZ. Cytokine coexpression during human Th1/Th2 cell differentiation: direct evidence for coordinated expression of Th2 cytokines. *J Immunol.* Sep 1 2002;169(5):2498-2506.
2. Neill DR, Wong SH, Bellosi A, et al. Nuocytes represent a new innate effector leukocyte that mediates type-2 immunity. *Nature.* 2010;464(7293):1367-1370.
3. Parmentier CN, Fuerst E, McDonald J, et al. Human TH2 cells respond to  cysteinyl leukotrienes through selective expression of cysteinyl leukotriene receptor 1. *Journal of Allergy and Clinical Immunology.* 2012;129(4):1136- 1142.

**Table E1.** Characteristics of CRSwNP patients and healthy volunteers.

|  | **CR** |  | **CRSwNP** | **Healthy** |
| --- | --- | --- | --- | --- |
| **Number of patients** |  |  | 20 | 7 |
|  |  |  |  |  |
| **Sex, number (%)** |  |  |  |  |
| Male |  |  | 10 (50) | 4 (57) |
| Female |  |  | 10 (50) | 3 (43) |
| **Age (year)** |  |  |  |  |
| Mean |  |  | 47 | 41 |
| Range |  |  | 34 - 65 | 20 - 53 |
| **Ethnicity, number (%)** |  |  |  |  |
| Caucasian |  |  | 17 (85) | 6 (86) |
| African-Caribbean |  |  | 2 (10) | 1 (14) |
| Asian |  |  | 1 (5) |  |
| **Co-morbidities, number (%)** |  |  |  |  |
| Asthma |  |  | 19 (95) | N.A. |
| Aspirin sensitivity |  |  | 13 (65) | N.A. |
| **Smoking status, number (%)** |  |  |  |  |
| Yes |  |  | 2 (10) | 1 (14) |
| **Skin prick test, number (%)** |  |  |  |  |
| Positive |  |  | 9 (45) | 0 (0) |
| Negative |  |  | 10 (50) | 7 (100) |
| Unknown |  |  | 1 (5) |  |
| **Steroids, number (%)** |  |  |  |  |
| Prednisolone (oral) |  |  | 6 (30) | N.A. |
| Flixonase (nasal) |  |  | 15 (75) | N.A. |
| **Other medication, number (%)** |  |  |  |  |
| Singulair |  |  | 5 (25) | N.A. |
| Cetirizine |  |  | 2 (10) | N.A. |

**Table E2.** Expression of T cell phenotype markers in CD4+ and CD8+ T cells derived from peripheral blood and polyp tissue (n = 14). Values shown are mean ± SEM. Wilcoxon matched-pairs signed rank test. ^≠^P<0.05 for CD4 polyp vs. CD4 blood; ^#^P<0.05 for CD8 polyp vs. CD8 blood.

|  | **Polyp** | | **Blood** | |
| --- | --- | --- | --- | --- |
| **Marker (%)** | **CD4+** | **CD8+** | **CD4+** | **CD8+** |
| CD45RO | 98.9 ± 0.8 | 94.7 ± 2.1 | ^≠^93.1 ± 2.8 | 85.2 ± 6.2 |
| TCRγδ | 4.7 ± 1.6 | 0.7 ± 0.3 | 4.4 ± 1.5 | 0.8 ± 0.2 |
| TCRαβ | 90.3 ± 2.2 | 80 ± 3.9 | 78.7 ± 5.9 | 75.7 ± 6.9 |
| CCR7 | 7.5 ± 3.5 | 3.5 ± 2.5 | ^≠^16.2 ± 4.4 | ^#^14.3 ± 4.6 |
| CD62L | 57.8 ± 4.1 | 26.9 ± 4.1 | ^≠^73.4 ± 6.8 | ^#^66.2 ± 8.3 |
| CD49a | 51.4 ± 5.6 | 80.2 ± 4.2 | ^≠^34.5 ± 6.9 | ^#^60.9 ± 5.0 |

Table E3. Differentially expressed genes in activated polyp-derived CD4+ IL-17RB+ and IL-17RB- populations, as determined by Illumina Human HT-12 v4 Expression BeadChip analysis of sorted cells. Genes are listed in order according to magnitude of difference in mRNA expression between IL-17RB+ vs. IL-17RB- T cells. P values were calculated using a 3 way-ANOVA model and significance indicated *.

| **Gene** | ***p*-value** | **Ratio** | **Fold-difference** |
| --- | --- | --- | --- |
| *IL17RB- (activated) down vs. IL17RB+ (activated)* | | | |
| IL5 | 0.008 * | 0.152 | -6.562 |
| IL17RB | 0.006 * | 0.191 | -5.225 |
| IL9 | 0.017 * | 0.278 | -3.601 |
| IL13 | 0.051 | 0.325 | -3.075 |
| GNS | 0.001 * | 0.335 | -2.989 |
| PTGS2 | 0.008 * | 0.343 | -2.917 |
| FANK1 | 0.007 * | 0.346 | -2.889 |
| DGKE | 0.003 * | 0.349 | -2.867 |
| GATA3 | 0.015 * | 0.389 | -2.568 |
| IL4 | 0.062 | 0.398 | -2.511 |
| CREG1 | 0.055 | 0.407 | -2.457 |
| PMCH | 0.011 * | 0.422 | -2.372 |
| PTPLA | 0.009 * | 0.440 | -2.274 |
| TSSC4 | 0.001 * | 0.469 | -2.133 |
| DIS3L | 0.075 | 0.473 | -2.116 |
| ARL6IP5 | 0.005 * | 0.484 | -2.067 |
| KLHL22 | 0.014 * | 0.495 | -2.018 |
| *IL17RB- (activated) up vs. IL17RB+ (activated)* | | | |
| RASGEF1A | 0.003 * | 2.001 | 2.001 |
| PLK3 | 0.012 * | 2.016 | 2.016 |
| OR7G3 | 0.002 * | 2.020 | 2.020 |
| SYT16 | 0.002 * | 2.025 | 2.025 |
| C10orf82 | 0.011 * | 2.027 | 2.027 |
| LOXHD1 | 0.018 * | 2.032 | 2.032 |
| PXDN | 0.003 * | 2.049 | 2.049 |
| PRR20D | 0.002 * | 2.056 | 2.056 |
| CLEC18C | 0.008 * | 2.065 | 2.065 |
| DGAT2 | 0.003 * | 2.078 | 2.078 |
| CCL4L1 | 0.180 | 2.095 | 2.095 |
| BAK1 | 0.004 * | 2.099 | 2.099 |
| FASLG | 0.021 * | 2.100 | 2.100 |
| EPX | 0.004 * | 2.139 | 2.139 |
| LTA | 0.196 | 2.193 | 2.193 |
| CCL3 | 0.054 | 2.203 | 2.203 |
| MDFI | 0.004 * | 2.258 | 2.258 |
| YKT6 | 0.034 * | 2.283 | 2.283 |
| SLC27A4 | 0.009 * | 2.343 | 2.343 |
| SUFU | 0.004 * | 2.372 | 2.372 |
| TOR3A | 0.012 * | 2.411 | 2.411 |
| PRF1 | 0.005 * | 2.489 | 2.489 |
| CCL3L1 | 0.015 * | 2.595 | 2.595 |
| IER3 | 0.017 * | 2.700 | 2.700 |
| IFNG | 0.055 | 3.032 | 3.032 |

**Table E4**. Differentially expressed genes in activated peripheral blood and healthy nasal mucosa derived CD4+ populations. Genes are listed in order according to magnitude of difference in mRNA expression between blood and nasal mucosal T cells. P values were calculated using a 3 way-ANOVA model and significance indicated *.

| **Gene** | ***p-*value** | **Ratio** | **Fold-difference** |
| --- | --- | --- | --- |
| *Blood (activated) down vs. normal nasal mucosa (activated)* | | | |
| IL17F | 2.41E-07 * | 0.068 | -14.672 |
| IL22 | 2.54E-06 * | 0.068 | -14.622 |
| CCL20 | 4.45E-06 * | 0.084 | -11.966 |
| KLRB1 | 7.17E-05 * | 0.110 | -9.087 |
| IL1R1 | 2.77E-05 * | 0.164 | -6.093 |
| IL2 | 1.40E-06 * | 0.189 | -5.301 |
| TNFSF8 | 2.74E-06 * | 0.199 | -5.013 |
| PCID2 | 5.10E-05 * | 0.211 | -4.731 |
| CDC42EP3 | 0.000153032 * | 0.243 | -4.123 |
| AIM2 | 3.46E-05 * | 0.250 | -4.000 |
| TNFSF9 | 2.44E-05 * | 0.256 | -3.911 |
| INS-IGF2 | 2.90E-05 * | 0.260 | -3.850 |
| VPS13C | 1.19E-06 * | 0.260 | -3.847 |
| IL26 | 0.000999067 * | 0.262 | -3.811 |
| C21orf71 | 4.20E-06 * | 0.274 | -3.649 |
| PTGER4 | 3.26E-05 * | 0.274 | -3.647 |
| MIR155HG | 0.000117239 * | 0.276 | -3.620 |
| GPR87 | 3.05E-05 * | 0.280 | -3.574 |
| PFKFB3 | 0.00013019 * | 0.282 | -3.547 |
| BATF3 | 0.000460554 * | 0.284 | -3.526 |
| FNBP1L | 3.25E-05 * | 0.288 | -3.468 |
| FREQ | 4.61E-06 * | 0.294 | -3.407 |
| STAT4 | 1.68E-05 * | 0.294 | -3.404 |
| ASCL2 | 4.43E-06 * | 0.294 | -3.397 |
| COL5A3 | 5.86E-06 * | 0.300 | -3.329 |
| NETO2 | 0.000253156 * | 0.312 | -3.202 |
| IQCG | 7.64E-05 * | 0.314 | -3.189 |
| TMEM88 | 7.80E-06 * | 0.314 | -3.184 |
| CTSL1 | 0.00131747 * | 0.318 | -3.145 |
| NCRNA00152 | 0.000345387 * | 0.319 | -3.132 |
| IL7R | 7.20E-05 * | 0.320 | -3.127 |
| IL1RL1 | 0.00254375 * | 0.327 | -3.054 |
| TIMP1 | 2.00E-05 * | 0.330 | -3.035 |
| MAP3K8 | 2.01E-06 * | 0.335 | -2.985 |
| CD44 | 3.96E-05 * | 0.341 | -2.935 |
| DUSP6 | 4.08E-05 * | 0.347 | -2.880 |
| BTG2 | 2.26E-05 * | 0.348 | -2.876 |
| CSF2 | 0.000256674 * | 0.350 | -2.860 |
| C2CD4B | 8.72E-07 * | 0.350 | -2.855 |

**FIGURE LEGENDS**

**Figure E1.** Comparison of T cell surface molecules expression by CD4+ cells derived from blood and nasal polyp explants. (a) Representative flow cytometry staining of CD4+ cells derived from matched blood and polyp tissue specimens. Cells were analyzed following anti-CD3/CD28 stimulation and expansion in IL-2 only for 7 days. Statistical analysis of data is shown in Table E2. (b) Representative staining for memory T cell markers CD62L and CCR7 in CD4+CD45RO+ cells derived from blood and nasal polyp explants. (c) Expression of CD62L and CCR7 in CD4+CD45RO+ cells from blood and polyp explants (n=13). Wilcoxon matched-pairs signed rank test.

**Figure E2**. Analysis of cytokine co-expression by polyp and blood-derived T cells. Representative flow cytometry staining is shown for the co-expression of IL-17 with IL-22 or IFNγ by CD4+ cells derived from matched blood and polyp tissue specimens. Three representative examples are shown with each row representing a single patient.

**Figure E3.** Th2 cytokine levels are elevated in polyp culture supernatants compared to normal nasal mucosal biopsy culture supernatants. Polyp (n=8) and normal nasal biopsy (NM bx)-derived cells (n=7) were stimulated with anti-CD3/CD28 antibodies for 24 hours before supernatants were harvested. Protein expression of IL-13, IL-5 and IL-4 was examined by CBA analysis. Mann Whitney test.

**Figure E4.** Comparison of polyp-derived CD4+ cells vs. CD8+ cells. (a) Representative flow cytometry staining of CD4+ and CD8+ T cells derived from matched blood and polyp tissue specimens. (b) T cell phenotype markers expressed by CD4+ (n=16) and CD8+ cells (n=10) from blood vs. polyp-derived tissue. Wilcoxon matched-pairs signed rank test.

**Figure E5.** Digested cultures show same phenotype as explant cultures. Polyp tissue was digested with collagenase overnight and PBMCs from the same donors were cultured in parallel. Cells were analyzed the following day by flow cytometry. Representative staining is shown in (a). Expression of IL-17RB (n = 4), IL-5, IL-13 and IL-17A in blood vs. polyp-derived cells (n=6) is shown in (b). Wilcoxon matched-pairs signed rank test.

**Figure E6.** RT-PCR validation of gene expression array data. RNA from sorted IL-17RB+/- cells was extracted. Expression of Th2 related genes was examined with TaqMan primer/probe sets following activation of cells with PMA/ionomycin for 4 hours. Wilcoxon matched-pairs signed rank test.

**Figure E7.** IL-25 is expressed by epithelium and eosinophils of nasal polyps. (a) Representative IHC staining for IL-25 in nasal polyp and normal nasal mucosal biopsies (x20 magnification). Control (IgG) is also shown. (b) Counts of IL-25+ cells per mm^2^ of tissue in the lamina propria of polyp vs. normal nasal biopsies (n = 9 per group). Mann Whitney test. (c) Representative IL-25 immunostaining in eosinophils within polyp tissue (x40 magnification).

**Figure E8.** IL-33 is expressed by epithelial and endothelial cells. Representative immunohistochemical staining for IL-33 in (a) epithelium and (b) endothelial cells of nasal polyp and normal nasal mucosal biopsies (x20 magnification). Control (IgG) is also shown.

**Figure E9.** Nasal mucosal and blood T cells show preferential gene expression. Heatmap shows hierarchical clustering of genes with significant differential expression (fold change >2 or <-2; and p<0.05) in sorted normal nasal mucosa- and blood-derived CD4+ T cells. Comparison of activated CD4+ normal nasal mucosal and activated CD4+ blood samples (n=3 per group) identified 301 genes that were differentially expressed. Th17-related genes are indicated.
